# Supplementary material for: AlphaFold-driven structural proteomics reveals extensive cellulosome machinery in human ruminococcal symbionts
Source: mBio. 2026 Jul 23;17(8):e01295-26. doi: 10.1128/mbio.01295-26 (PMC13463774; doi:10.1128/mbio.01295-26)
Supplement: Supplementary file 1 [file mbio.01295-26-s0001.docx]

**Supplementary Figures and Tables**

**Figure S1. Sequence identified cohesin domains across cellulosome producing species.** Cohesin and dockerin domain counts in representative cellulosome-producing bacteria from the *Acetivibrio* (*Acetivibrio thermocellus*), *Ruminoclostridium* (*Ruminoclostridium cellulolyticum*), *Clostridium* (*Clostridium acetobutylicum*), and *Ruminococcus* (*Ruminococcus callidus*) genera. Cohesin and dockerin counts shown are determined from InterProScan sequence profiles (Cohesins: type I - cd08548, type II - cd08547, type III - cd08759, cohesin domain - PF00963; Dockerins: type I - cd14256, type II - cd14254, type III - cd14255).

**Figure S2. Established TM-score threshold is consistent across other cellulosomal *Ruminococcus*. (A)** Representative plots showing TM-score versus domain count and **(B)** RMSD α-carbon coordinate difference versus number of aligned α-carbon atoms for predicted structures in *Ruminococcus flavefaciens* (top) and *R. champanellensis* (bottom). 6845 and 4922 individual domains were compared to a type I cohesin (PDB: 1OHZ) for *R. flavefaciens* and *R. champanellensis*, respectively. The threshold TM-score used for cohesin domain identification is shown as a dashed red line that separates a large low-scoring population of unrelated structures (left) from a high-scoring structurally related population (right).

**Figure S3. AlphaFold identifies a large number of novel cohesin domains in *Ruminococcus* proteomes.** **(A)** Bar graph showing the total number of cohesin domains identified per *Ruminococcus* proteome. Total bar height represents the number of cohesin domains identified across all annotation methods used (AF2, InterProScan, BLAST). Segments colored purple indicate AF2-only detected cohesins. Also shown are counts for representative non-*Ruminococcus* cellulosome producing bacteria from the *Acetivibrio* (*Acetivibrio thermocellus*), *Ruminoclostridium* (*Ruminoclostridium cellulolyticum*), and *Clostridium* (*Clostridium acetobutylicum*) genera. **(B)** Venn diagram of the total number of cohesin domains identified by AF2 (pink), InterProScan (cyan), and BLAST (yellow) in analyzed *Ruminococcus* proteomes.

**Figure S4. Structure-based dendrogram of experimentally determined cohesin structures.** Structural dendrogram of all 52 experimentally determined cohesin atomic structures deposited in the PDB. The dendrogram was constructed from an all-on-all TM-score matrix. The majority of structures can be classified into structural groups 1 to 3 whose members are frequently assigned to type I to III cohesin families based on their primary sequences, respectively. In the figure these distinct primary sequences are indicted with dots (type I (yellow, cd08548), type II (cyan, cd08547), type III (red, cd08759), and un-typed (black, PF00963). Additional, sparsely populated structural families are also formed by cohesins from *R. bromii*, *C. perfringens*, and *R. flavefaciens* species. The minimum TM-score for inclusion into groups 1 to 3 is indicated.

**Figure S5**. **Sequence Similarity Network (SSN) of 177 AF2-only identified ruminococcal cohesins**. Sequence similarity network (SSN) of 177 AF2-only identified cohesins was created by EFI-EST and visualized with CytoScape. Three additional sequences are included as representative type I, II, and III cohesins from the PDB (type-1: 1OHZ, type-2: 1TYJ, type-3: 2ZF9). A sequence similarity cutoff of 30% was used to generate clusters. Colors of nodes denote the structural group each cohesin domain belongs to; Yellow: Group1, Green: Group 4, Purple: Group 5, Pink: Group 6, Grey: Group7, Orange: No assigned group.

**Figure S6. Crystal structures of novel *R. callidus* cohesins.** Cartoon models of experimentally determined structures for six additional putative cohesins in *R. callidus* scaffoldin-like proteins. Overlays of the crystal structure shown here and the corresponding predicted AlphaFold2 (purple) model are shown on the right with the calculated backbone root mean square deviation (RMSD) between the two structures.

**Figure S7. Cellulosome-like architectures in non-human associated Ruminococci**. Cartoon schematic of the cellulosomal proteins in two species analyzed that did not follow the archetypes of other *Ruminococcus* cellulosomes established in **Fig. 5**, *Ruminococcus bromii* and *Ruminococcus* sp. HUN007. Color and shading for each cohesin domain follows the same convention established in **Fig. 5**. Along with those represented in **Fig. 5**, these cartoon schematics represent all cellulosome-like assemblies discussed, with the exception of *Ruminococcus albus*. Additional domains within the scaffoldin and scaffoldin-like proteins are also indicated: **LRR** - Leucine Rich Repeat, **αAmy** - α-Amylase, **CBM26** - Carbohydrate Binding Module Family 26, **Flg** - Listeria-Bacteroides repeat domain, **Endopep** - endopeptidase domain, **SH3** - SRC Homology 3 domain, **Ricin** - Ricin B-like lectin, **X** - X2 domain, **GH** - glycoside hydrolase, **CAP** - Cysteine-rich secretory / Antigen 5 / Pathogenesis-related 1 domain.

**Figure S8. Cellulolytic capacity of ruminococcal species.** Counts for DocGH and Dockerin-containing proteins are plotted for species within each cellulosome-archetype. Those species outlined in black represent human-gut associated microbes and those in grey are non-human gut associated. The unclassified species are *Ruminococcus albus* (58, 12) and *Ruminococcus HUN007* (57, 222) which has a unique cellulosome architecture.

**Table S1:** ***Ruminococcus spp.* Genome metrics.** List of the genome quality statistics for all analyzed *Ruminococcus* genomes including species name, GCF reference, taxonomy ID, isolation location, assembly statistics, and associated software packages used to analyze the genome. The N50 contig value is the shortest length contig that when added with all other contigs greater in length sums to roughly 50% of the total assembly length. All analyzed *Ruminococcus* genomes were annotated using Prokka.^55^ A * symbol indicates *Ruminococcus* species listed on the List of Prokaryotic Names with Standing in Nomenclature. In our analysis, we also inspected the genomes of several species that were originally assigned as *Ruminococcus* bacteria, but have since been reassigned to *Blautia*, *Mediterraneibacter*, *Trichococcus*, and *Hominimerdicola* genera. The additional nine genomes that were analyzed were not found to have cellulosome-like structures after AF analysis: *R. sp._RGIG8444, R. intestinalis, R. sp. BSD2780120874_150323_B10, R. equi, R. sp. FMB-CY1, R. sp. JL13D9, R. sp. YE71, R. sp. YE78, R. bovis, R. sp. FC2018*.

| **Protein ID** | **Group** | **Identification method** | | **AF2 / TM-align** | | | **Reference** |
| --- | --- | --- | --- | --- | --- | --- | --- |
|  |  | **BLAST** | **AF2** | **Type I  (1OHZ)** | **Type II (1TYJ)** | **Type III (2ZF9)** |  |
| WP_014522594 | 1 | ✓ | ✓ | 0.98 | 0.67 | 0.64 |  |
|  | 1 | ✓ | ✓ | 0.98 | 0.67 | 0.63 | 34 |
|  | 1 | ✓ | ✓ | 0.97 | 0.69 | 0.65 |  |
|  | 1 | ✓ | ✓ | 0.97 | 0.69 | 0.65 |  |
|  | 1 | ✓ | ✓ | 0.97 | 0.69 | 0.65 | 35 |
|  | 1 | ✓ | ✓ | 0.96 | 0.70 | 0.64 |  |
| WP_014522595 | 2 | ✓ | ✓ | 0.77 | 0.88 | 0.62 |  |
|  | 2 | ✓ | ✓ | 0.77 | 0.89 | 0.64 |  |
|  | 2 | ✓ | ✓ | 0.76 | 0.89 | 0.62 |  |
|  | 2 | ✓ | ✓ | 0.77 | 0.89 | 0.63 |  |
| WP_003519375 | 2 | ✓ | ✓ | 0.77 | 0.90 | 0.62 |  |
|  | 2 | ✓ | ✓ | 0.77 | 0.88 | 0.63 | 36 |
| WP_003519374 | 1 | ✓ | ✓ | 0.94 | 0.70 | 0.64 |  |
| WP_003516982 | 2 | ✓ | ✓ | 0.78 | 0.86 | 0.63 | 37 |
| WP_003516263 | 2 | ✓ | ✓ | 0.79 | 0.90 | 0.63 |  |
|  | 2 | ✓ | ✓ | 0.79 | 0.91 | 0.64 |  |
|  | 2 | ✓ | ✓ | 0.79 | 0.92 | 0.65 |  |
|  | 2 | ✓ | ✓ | 0.80 | 0.91 | 0.64 |  |
|  | 2 | ✓ | ✓ | 0.78 | 0.90 | 0.64 |  |
|  | 2 | ✓ | ✓ | 0.78 | 0.89 | 0.63 |  |
|  | 2 | ✓ | ✓ | 0.77 | 0.89 | 0.62 |  |
| WP_003516262 | 2 | ✓ | ✓ | 0.78 | 0.89 | 0.63 |  |
| WP_235825069 | 1 | ✓ | ✓ | 0.92 | 0.69 | 0.69 | 38 |

**Table S2. Comparison of cohesins detected by AF2 and BLAST for *Clostridium thermocellum.*** Tabulation of putative cohesin domain-containing scaffoldin proteins for *C. thermocellum* including; protein accession code (Protein ID), cohesin structural group (Group), identification method (BLAST or AF2; identified by ‘✓’ and ‘X’), TM-align scores to each representative cohesin domain (AF2 / TM-align), and relevant references.

| **Protein ID** | **Group** | **Identification method** | | **AF2 / TM-align** | | | **Reference** |
| --- | --- | --- | --- | --- | --- | --- | --- |
|  |  | **BLAST** | **AF2** | **Type I  (1OHZ)** | **Type II (1TYJ)** | **Type III (2ZF9)** |  |
| WP_015924274 | 1 | ✓ | ✓ | 0.90 | 0.82 | 0.83 | 39 |
|  | 1 | ✓ | ✓ | 0.90 | 0.82 | 0.83 | 40 |
|  | 1 | ✓ | ✓ | 0.90 | 0.82 | 0.83 | 40 |
|  | 1 | ✓ | ✓ | 0.90 | 0.82 | 0.83 | 40 |
|  | 1 | ✓ | ✓ | 0.90 | 0.82 | 0.83 |  |
|  | 1 | ✓ | ✓ | 0.90 | 0.82 | 0.83 |  |
|  | 1 | ✓ | ✓ | 0.90 | 0.82 | 0.84 |  |
|  | 1 | ✓ | ✓ | 0.91 | 0.84 | 0.83 |  |
| WP_015924279 | 1 | ✓ | ✓ | 0.89 | 0.81 | 0.83 |  |
| WP_015925035 | 2 | ✓ | ✓ | 0.75 | 0.78 | 0.75 |  |

**Table S3. Comparison of cohesins detected by AF2 and BLAST for *Ruminiclostridium cellulolyticum.*** Tabulation of putative cohesin domain-containing scaffoldin proteins for *R. cellulolyticum* including; protein accession code (Protein ID), cohesin structural group (Group), identification method (BLAST or AF2; identified by ‘✓’ and ‘X’), TM-align scores to each representative cohesin domain (AF2 / TM-align), and relevant references.

| **Protein ID** | **Group** | **Identification method** | | **AF2 / TM-align** | | | **Reference** |
| --- | --- | --- | --- | --- | --- | --- | --- |
|  |  | **BLAST** | **AF2** | **Type I  (1OHZ)** | **Type II (1TYJ)** | **Type III (2ZF9)** |  |
| WP_010964232 | 1 | ✓ | ✓ | 0.90 | 0.69 | 0.64 |  |
| WP_010964228 | 1 | ✓ | ✓ | 0.88 | 0.69 | 0.64 |  |
|  | 1 | ✓ | ✓ | 0.88 | 0.69 | 0.64 | 41 |
|  | 1 | ✓ | ✓ | 0.88 | 0.69 | 0.65 |  |
|  | 1 | ✓ | ✓ | 0.88 | 0.70 | 0.64 |  |
|  | 1 | ✓ | ✓ | 0.87 | 0.68 | 0.64 |  |

**Table S4. Comparison of cohesins detected by AF2 and BLAST for *Clostridium acetobutylicum.*** Tabulation of putative cohesin domain-containing scaffoldin proteins for *C. acetobutylicum* including; protein accession code (Protein ID), cohesin structural group (Group), identification method (BLAST or AF2; identified by ‘✓’ and ‘X’), TM-align scores to each representative cohesin domain (AF2 / TM-align), and relevant references.

**Table S5. X-ray data-collection and refinement statistics for *R. callidus* cohesins: ***Values in parentheses are for the highest resolution shell.

**Table S6. Predicted enzyme content of cellulosome displaying bacteria:** Dockerin-enzyme content of 33 *Ruminococcus* species grouped together within their phylogenetic relationships. Total dockerin proteins, cellulase-doc, hemicellulase-doc, polymer-active DocGH (combined cellulase and hemicellulase-DocGH content), and CAZyme-Doc proteins are noted. The dockerin-enzyme profile is divided into protein counts of dockerin-fused amylase, cellulase, and hemicellulase active enzymes. *CBM26 is a starch binding carbohydrate binding module present in *Ruminococcus* species that contain Amylase-Doc proteins.

| **Protein ID** | **Gene locus** | **Group** | **AF2 / TM-align** | | | **InterProScan** | | **BLAST**  **identified** | **Modular arrangement** |
| --- | --- | --- | --- | --- | --- | --- | --- | --- | --- |
|  |  |  | **Type I**  **(1OHZ)** | **Type II**  **(1TYJ)** | **Type III**  **(2ZF9)** | **CDD** | **Pfam** |  |  |
| WP_021681153 | Rcal_0005  (ScaE-like) | 4 | 0.71 | 0.62 | 0.61 | - | - | - | SIGN-**Coh**-LPXTG |
| WP_021682353 | Rcal_0152  (ScaC-like) | 1 | 0.87 | 0.70 | 0.68 | cd08548 | PF00963 | ✔ | SIGN-**Coh**-Doc |
| WP_370809552 | Rcal_0153  (ScaB-like) | 6 | 0.71 | 0.67 | 0.64 | - | - | - | SIGN-**Coh**-**Coh**-**Coh**-**Coh**-X-Doc |
|  |  | 5 | 0.79 | 0.70 | 0.68 | - | - | - |  |
|  |  | 5 | 0.79 | 0.71 | 0.68 | - | - | - |  |
|  |  | 5 | 0.79 | 0.69 | 0.65 | - | - | - |  |
| WP_051343558 | Rcal_0670 | 5 | 0.76 | 0.70 | 0.69 | - | - | - | SIGN-DUF5620--**Coh**-**Coh**-- |
|  |  | 5 | 0.76 | 0.68 | 0.65 | - | - | - |  |
| WP_021682326 | Rcal_1096 | 7 | 0.54 | 0.44 | 0.41 | - | PF00963 | ✔ | SIGN-**Coh** |
| WP_021680365 | Rcal_2513 | 6 | 0.73 | 0.67 | 0.63 | - | - | - | SIGN-**Coh**-- |
| WP_169725972 | Rcal_2938 | 4 | 0.70 | 0.59 | 0.60 | - | - | - | SIGN-CAP-**Coh**-**Coh**-Doc |
|  |  | 4 | 0.71 | 0.61 | 0.62 | - | - | - |  |
| WP_021680502 | Rcal_2942 | 4 | 0.76 | 0.64 | 0.64 | - | - | - | SIGN-**Coh**-**Coh**-**Coh**-**Coh**- Doc |
|  |  | 4 | 0.76 | 0.63 | 0.63 | - | - | - |  |
|  |  | 4 | 0.77 | 0.66 | 0.66 | - | - | - |  |
|  |  | 4 | 0.77 | 0.64 | 0.63 | - | - | - |  |

**Table S7. Putative Scaffoldin proteins and cohesin domains identified in *Ruminococcus callidus***: Tabulation of putative cohesin domain-containing scaffoldin proteins for *R. callidus* including; protein accession code (Protein ID), Gene Locus, cohesin structural group (Group), TM-align scores to each representative cohesin domain (AF2 / TM-align), InterProScan sequence identifier annotation (CDD and Pfam), if the domain was identified by BLAST (BLAST Identified), and domain arrangement according to structurally and sequence-identified domains (Modular Arrangement). Abbreviations: cohesin (**Coh**), dockerin (**Doc**), cysteine-rich secretory / antigen 5 / pathogenesis-related 1 domain (**CAP**), sortase sorting signal (**LPXTG**), domain of unknown function (**DUF5620**), signal peptide (**SIGN**)**,** X-module (**X**).

| **Protein ID** | **Gene locus** | **Group** | **AF2 / TM-align** | | | **InterProScan** | | **BLAST**  **identified** | **Modular arrangement** |
| --- | --- | --- | --- | --- | --- | --- | --- | --- | --- |
|  |  |  | **Type I**  **(1OHZ)** | **Type II**  **(1TYJ)** | **Type III**  **(2ZF9)** | **CDD** | **Pfam** |  |  |
| WP_186832788 | JKK62_RS06065 | 7 | 0.69 | 0.58 | 0.54 | - | PF00963 | ✔ | SIGN-**Coh**--TMH- |
| WP_201426792 | JKK62_RS02320 | 1 | 0.76 | 0.63 | 0.63 | - | PF00963 | ✔ | SIGN-LRR---**Coh**- Doc |
| WP_201426793 | JKK62_RS02325 | 1 | 0.80 | 0.65 | 0.62 | - | PF00963 | ✔ | SIGN-**Coh**-Doc |
| WP_201426884 | JKK62_RS02780 | 7 | 0.69 | 0.61 | 0.55 | - | - | - | SIGN-**Coh**-**Coh**-**Coh**-**Coh**-**Coh**-Doc |
|  |  | 7 | 0.69 | 0.60 | 0.57 | - | - | - |  |
|  |  | 7 | 0.68 | 0.60 | 0.54 | - | - | - |  |
|  |  | 7 | 0.70 | 0.61 | 0.56 | - | - | - |  |
|  |  | 7 | 0.69 | 0.60 | 0.55 | - | - | - |  |
| WP_201427080 | JKK62_RS04600 | 7 | 0.69 | 0.61 | 0.55 | - | - | - | SIGN-Doc-**Coh**-**Coh**-**Coh**-CBM26- |
|  |  | 7 | 0.69 | 0.61 | 0.56 | - | - | - |  |
|  |  | 7 | 0.70 | 0.61 | 0.55 | - | - | - |  |
| WP_201427120 | JKK62_RS06070 | 7 | 0.64 | 0.56 | 0.53 | - | - | - | SIGN-**Coh**-**Coh**-Ig- **Coh**-**Coh**-**Coh**-**Coh**- TMH |
|  |  | 7 | 0.69 | 0.60 | 0.55 | - | - | - |  |
|  |  | 7 | 0.70 | 0.61 | 0.55 | - | - | - |  |
|  |  | 7 | 0.69 | 0.61 | 0.55 | - | - | - |  |
|  |  | 7 | 0.70 | 0.58 | 0.54 | - | - | - |  |
|  |  | 7 | 0.68 | 0.59 | 0.56 | - | - | - |  |
| WP_201427518 | JKK62_RS08240 | 7 | 0.67 | 0.58 | 0.55 | - | - | ✔ | SIGN-**Coh**- |
| WP_201427542 | JKK62_RS08470 | 1 | 0.78 | 0.66 | 0.63 | - | PF00963 | ✔ | SIGN-LRR---**Coh**- Doc |
| WP_201427543 | JKK62_RS08475 | 1 | 0.79 | 0.66 | 0.64 | - | PF00963 | ✔ | SIGN-**Coh**-Doc |
| WP_201427787 | JKK62_RS10155 | 7 | 0.66 | 0.58 | 0.55 | - | - | - | SIGN-**Coh** |
| WP_201427966 | JKK62_RS17810 | 7 | 0.68 | 0.59 | 0.55 | - | - | - | SIGN-Doc-**Coh**-**Coh**--**Coh**-**Coh**-**Coh**- CBM26-CBM26 |
|  |  | 7 | 0.68 | 0.60 | 0.56 | - | - | - |  |
|  |  | 7 | 0.68 | 0.61 | 0.56 | - | - | - |  |
|  |  | 7 | 0.69 | 0.61 | 0.56 | - | - | - |  |
|  |  | 7 | 0.68 | 0.61 | 0.55 | - | - | - |  |
| WP_201428926 | JKK62_RS16760 | 7 | 0.65 | 0.56 | 0.53 | cd08547 | - | ✔ | SIGN-**Coh**-**Coh**-**Coh**--Doc |
|  |  | 7 | 0.67 | 0.57 | 0.54 | cd08547 | - | ✔ |  |
|  |  | 7 | 0.65 | 0.57 | 0.54 | - | - | ✔ |  |

**Table S8. Putative Scaffoldin proteins and cohesin domains identified in *Ruminococcus difficilis*:** Tabulation of putative cohesin domain-containing scaffoldin proteins for *R. difficilis* including; protein accession code (Protein ID), Gene Locus, cohesin structural group (Group), TM-align scores to each representative cohesin domain (AF2 / TM-align), InterProScan sequence identifier annotation (CDD and Pfam), if the domain was identified by BLAST (BLAST Identified), and domain arrangement according to structurally and sequence-identified domains (Modular Arrangement). Abbreviations: cohesin (**Coh**), dockerin (**Doc**), carbohydrate binding module, family 26 (**CBM26**), predicted transmembrane helix region (**TMH**), leucine-rich repeat region (**LRR**), signal peptide (**SIGN**)
